# Time-keeping and decision-making in living cells: Part I

**DOI:** 10.1098/rsfs.2022.0011

**Published:** 2022-04-15

**Authors:** John J. Tyson, Attila Csikasz-Nagy, Didier Gonze, Jae Kyoung Kim, Silvia Santos, Jana Wolf

**Affiliations:** ^1^ Department of Biological Sciences, Virginia Polytechnic Institute and State University, Blacksburg, VA 24061, USA; ^2^ Faculty of Information Technology and Bionics, Pázmány Péter Catholic University, 1088 Budapest, Hungary; ^3^ Unit of Theoretical Chronobiology, Université Libre de Bruxelles, 1050 Brussels, Belgium; ^4^ Department of Mathematical Sciences, KAIST, Daejeon 34141, South Korea; ^5^ Biomedical Mathematics Group, Institute for Basic Science, Daejeon 34126, South Korea; ^6^ Quantitative Stem Cell Biology Laboratory, The Francis Crick Institute, London NW1 1AT, UK; ^7^ Mathematical Modeling of Cellular Processes, Max Delbrück Center for Molecular Medicine, 13125 Berlin, Germany; ^8^ Department of Mathematics and Computer Science, Free University, 14195 Berlin, Germany

**Keywords:** molecular regulatory networks, feedback loops, mathematical modelling, multi-rhythmicity, entrainment, circadian rhythms

## Abstract

To survive and reproduce, a cell must process information from its environment and its own internal state and respond accordingly, in terms of metabolic activity, gene expression, movement, growth, division and differentiation. These signal–response decisions are made by complex networks of interacting genes and proteins, which function as biochemical switches and clocks, and other recognizable information-processing circuitry. This theme issue of *Interface Focus* (in two parts) brings together articles on time-keeping and decision-making in living cells—work that uses precise mathematical modelling of underlying molecular regulatory networks to understand important features of cell physiology. Part I focuses on time-keeping: mechanisms and dynamics of biological oscillators and modes of synchronization and entrainment of oscillators, with special attention to circadian clocks.

## 1. Introduction

**Figure 1.**
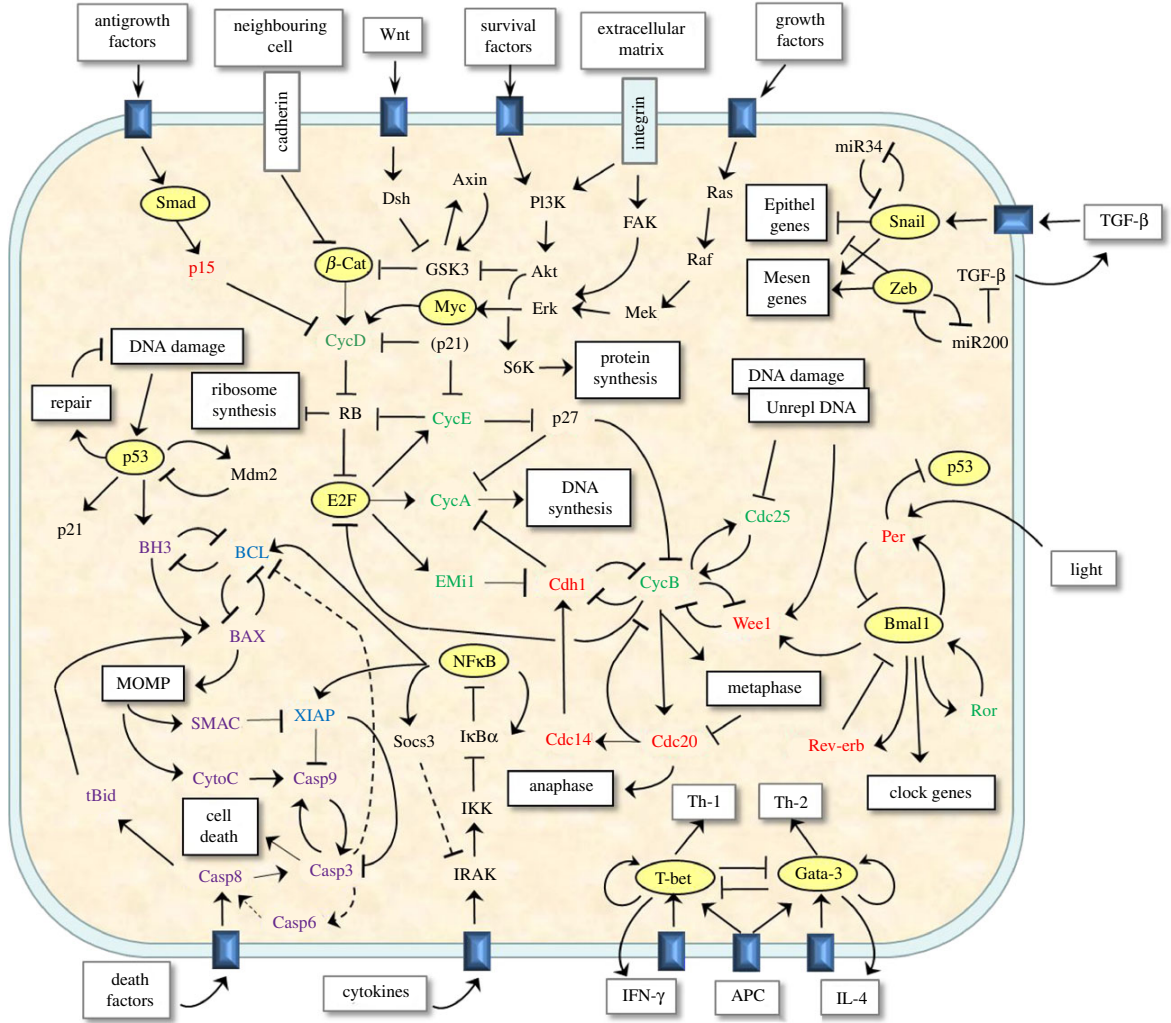
Some components of the information-processing system (IPS) in a mammalian cell. External signals (e.g. growth factors, death factors, cytokines, etc.) and internal signals (e.g. DNA damage, unreplicated DNA, mitochondrial outer membrane permeability—MOMP) are decoded through a cell's IPS—a complex network of interacting genes and proteins—to determine the best course of action for the cell given this information. Appropriate cellular responses include cell growth and division, cell differentiation, phase resetting of the circadian clock, cell cycle arrest, damage repair or cell death. The signals along the top provide input to cell growth (ribosome synthesis and protein synthesis) and division (DNA synthesis, mitosis). To the left, we see the DNA-damage response, mediated by p53, culminating (if it cannot be repaired) in cell death (apoptosis). Bottom left is the extrinsic cell death response through Caspase 8. Bottom middle is cytokine activation of the master transcription factor NF*κ*B. Bottom right is one example of T-helper cell differentiation, mediated by the transcription factors T-bet and Gata-3. On the lower right is the core circadian clock (Per/Bmal1/Rev-erb/Ror), and the upper right is a schematic of the epithelial/mesenchymal differentiation decision pathway.

Living cells are remarkably effective and adaptable information-processing systems (IPSs) that receive information from the cell's external environment and internal conditions, integrate concurring and conflicting signals, figure out appropriate responses and induce their implementation (figure 1). As in a digital computer, cellular IPSs rely on the dynamics of molecular ‘switches' and biological ‘clocks', but rather than solid-state electronic devices, these biochemical switches and clocks are made up of genes, proteins and metabolites interacting by reaction, diffusion and transport in a tiny volume (approx. 10^−12^ l) of gel-like cytoplasm [1–5]. Cellular IPSs are autonomous, analogue and massively parallel and often knocked about by stochastic fluctuations among the limited numbers of molecules in such small volumes. Nonetheless, their responses are remarkably successful in supporting the survival, growth, repair and reproduction of cells. A grand challenge of molecular systems biology is to understand how cellular IPSs work (basic science), how to fix them when they malfunction (health science) and how we might re-engineer them to our specifications (biotechnology).

The theoretical foundation for understanding time-keeping and decision-making in molecular regulatory networks was crafted in the 1960s and early 1970s by the pioneering work of Goodwin [6], Higgins [7], Griffith [8,9], Roessler [10], Thomas [11] and others. Over the next 30 years, much progress was made in uncovering design principles of cellular control systems and carrying out novel experimental tests of theoretical ideas. This history has been documented in great detail in research monographs by Segel [12], Goldbeter [13], Alon [14], Keener & Sneyd [15] and Ferrell [16] and briefly by Tyson *et al*. [17] in a supplemental issue of *Journal of the Royal Society Interface* on ‘biological switches and clocks'. As a follow-up to that issue, *Interface Focus* is presenting a two-part collection of articles on the theme of ‘time-keeping and decision-making in living cells'.

Part I focuses on time-keeping, in particular on mechanisms of biological oscillators, on synchronization of intercommunicating oscillators and on entrainment to external driving rhythms, with particular emphasis on circadian rhythms. Jiménez *et al.* [18] lead off the collection with a valuable survey of entrainment among biological oscillators, focusing on six representative examples: the circadian clock, the cell cycle, mitotic exit (Cdc14 endocycles), cardiac pacemaker cells (Ca^2+^/cyclic AMP (cAMP) oscillations), glycolytic oscillations and inflammatory responses (nuclear factor-κB (NFκB) oscillations). Next, Goldbeter & Yan [19] present a masterly review of multi-rhythmicity (two or more simultaneously stable oscillatory states in models of biochemical reaction networks) and multi-synchronization (two or more simultaneously stable modes of synchronization of coupled biological oscillators). Examples are drawn from cAMP signalling, circadian rhythms and cell cycle oscillations. Burckard *et al.* [20] provide new results on the synchronization of peripheral circadian clocks by intercellular communication between two cells or in small clusters of cells. In the final contribution, Jeong *et al.* [21] investigate the role of multiple modes of transcriptional repression in generating many of these rhythms. By modelling three mechanisms of transcriptional repression—repressor R may bind to activator A on promoter P to block transcription (R:A:P is transcriptionally inactive), R binding to A may displace A from the promoter (R + A:P → R:A:P → R:A + P) and R binding to A may sequester A from P (R:A prevents A from binding to P)—they show that synergistic interactions of the three modes generate ultrasensitive transcriptional responses and robust oscillations.

Part II will focus on decision-making in cell differentiation, development and cell cycle progression.

## 2. Other recent developments in time-keeping

Because this theme issue presents only a few snapshots of the immense progress that has been made on biological clocks since the 2008 collection, we review here some other recent developments.

As might be expected, considerable progress has been made in understanding the molecular mechanisms underlying circadian rhythms. Firstly, some new models of mammalian clock, taking into account interactions among the principal clock genes (*Per1*, *Per2*, *Cry1*, *Cry2*, *Clock*, *Bmal1*, *Rev-erbα* and *Rorα*), have been remarkably successful in accounting for the physiological properties of circadian rhythms in wild-type and mutant cell lines [22–24]. These mathematical models have proven their usefulness in applications to chronotherapy [25–28], wearable devices [29,30] and diagnostic tools [31,32]. The development of multi-scale circadian models, linking intra- and intercellular dynamics, has been pursued by DeWoskin *et al*. [33,34]. Other theoretical studies have revealed some general features of the molecular architecture of circadian clocks. Maeda & Kurata [35] showed that the dual-feedback structure found in living organisms supports a robust and entrainable oscillator. The roles of feedback and other design principles in the robustness of oscillations were analysed in many studies (e.g. Kim & Forger [24], Tsai *et al*. [36], Hafner *et al*. [37], Ananthasubramaniam [38] and Baum *et al*. [39]). By fitting models to experimental data, Herzel and co-workers [40,41] identified the important control loops at play in the suprachiasmatic nucleus (SCN) and peripheral organs. In particular, they have identified a three-component negative feedback loop (*Cry1* −| *Per2* −| *Rev-erbα* −| *Cry1*) as a dominant source of oscillations in their models [42]. Ko *et al*. [43] have highlighted the possible role of noise in generating self-sustained circadian rhythms. Notably, the mystery of temperature compensation of the circadian clock has been cleared up by a recognition of the temperature-dependent properties of the phospho-switch that regulates PERIOD-2 (PER2) protein phosphorylation by casein kinase-1 and its subsequent degradation, after polyubiquitination by beta-transducin repeats-containing protein (βTrCP) [44–46].

Intercellular coupling and synchronization of circadian clocks have also been the subject of many publications. Bernard *et al*. [47] showed that robust, self-sustained circadian oscillations can emerge from a heterogeneous network of damped oscillators through intercellular coupling. Hafner *et al*. [37] suggested that heterogeneity of circadian clocks in the SCN decreases the sensitivity of the network to brief perturbations while simultaneously improving its adaptation to long-term entrainment signals. Recent studies found that the heterogeneous responses of master and slave clock neurons to entrainment signals are critical for strong, adjustable circadian rhythms [48]. Studies by Vasalou *et al.* [49,50] and by Ananthasubramaniam *et al*. [51] have focused on the roles of neurotransmitters in synchronizing circadian oscillators in the SCN. Meanwhile, Webb *et al*. [52] further investigated the synchronization of weak, heterogeneous oscillators to produce robust circadian rhythms.

The interplay of circadian clocks and metabolism has received much attention. Using a combination of mathematical modelling and experiments in *Neurospora crassa*, Dovzhenok *et al*. [53] found an additional negative feedback loop that maintains circadian period over a wide range of glucose conditions. Woller *et al*. [54] introduced metabolic sensors into a mathematical model of the mammalian circadian clock to study the effects of diet (normal, high-fat, fasting) on clock function. Woller & Gonze [55] used a clock model to show how conflicting zeitgebers (light and food intake) disrupt the phase relations of core clock genes, leading to metabolic complications such as hyperglycaemia. Bae & Androulakis [56,57] developed models to study the impact of the circadian clock on insulin secretion and gluconeogenesis, showing how external signals (light/dark cycles and feeding/fasting cycles) affect metabolism over the course of a day. In a different vein, Rao *et al*. [58] used a model of the hypothalamus–-pituitary–adrenal axis and the sleep/wake cycle to explore the influence of sleep deficiency on daily rhythms of cortisol release.

We also draw your attention to some additional studies of interactions between the circadian clock and the cell division cycle, based on the unexpected discovery by Matsuo *et al*. [59] that transcription of the cell cycle gene *Wee1* is upregulated by an E-box, which binds to the master transcription factor BMAL1 : CLOCK. Zamborszky *et al*. [60] used mathematical modelling to show how the circadian clock, by gating *Wee1* expression, would generate quantized cell cycle times and cell division sizes. Later, Gerard & Goldbeter [61] found entrainment bands (at 24 h and 48 h) for a cell cycle model (without size control) driven by a 24 h rhythm of BMAL1 : CLOCK. On the experimental side, Feillet *et al*. [62] found multiple modes of entrainment and phase locking between cell divisions and the circadian rhythms in mouse fibroblasts whose clock phases were synchronized by a pulse of dexamethasone, which can be observed and modelled also on the single cell level [63]. And, Matsu-Ura *et al*. [64], using dual luciferase reporters for cell cycle and circadian oscillators, observed 2 : 1 entrainment (i.e. 12 h cell division cycles) in mouse intestinal organoids. In a different vein, Gotoh *et al*. [65] used mathematical models to gain insight into the coupling of the circadian clock to the DNA damage checkpoint (i.e. the interactions between PER2 and p53 proteins).

Finally, we review recent proposals that the cell division cycle is a ‘clock-shop' of autonomous (independent) oscillators entrained to one another. It is commonly thought that cell cycle events (DNA replication, mitosis, cell division) are controlled by a ‘master programme' based on fluctuations of cyclin-dependent kinases (Cdks) and their immediate regulators [66,67]. However, it is now established that many cell cycle events continue in a repetitive fashion even when the Cdk programme is blocked by mutations. For example, periodic processes that continue more-or-less on schedule in mutant (non-dividing) strains of budding yeast include budding and mating projections [68], DNA endoreplication [69], spindle pole body re-duplication [70], gene transcription [71], Cdc14 release from the nucleolus (a marker of mitotic exit) [72,73] and metabolic oscillations (NADPH fluctuations) [74]. Also, periodic centriole biogenesis (Polo kinase-4 oscillations) has been observed in mutant fruit fly embryos [75]. Furthermore, chromosome endoreduplication [76] and basal body (centriole) amplification [77] are characteristics of certain terminally differentiated (non-dividing) cells. These observations support the ‘clock-shop' hypothesis [71,72,78], although there are opposing opinions [73,79,80]. In the future, we may expect continued debate on the possible roles of ‘autonomous clocks' in cell cycle progression.

Altogether, these recent studies and many others have contributed greatly to our understanding of the molecular regulatory mechanisms underlying biological oscillations and of their advantageous properties, such as robustness, tunability, entrainment and temperature compensation. Also, we now have a better appreciation of the—often non-intuitive—dynamics resulting from the interplay between clocks and clock-controlled processes. This progress has revolutionized our interpretation of experimental observations and our vision of future progress in health science and biotechnology.

## 3. In memoriam

The theoretical foundations of time-keeping and decision-making by the molecular regulatory networks in living cells were laid out in the 1970s and 1980s by a small band of physical chemists, biochemists, chemical engineers and mathematical biologists. Unfortunately, many of these pioneering scientists have passed away and are sorely missed. In conclusion, we would like to recognize their contributions to the field.

Joel E. Keizer (1942–1999). As a physical chemist, Joel Keizer made fundamental contributions to the theory of non-equilibrium thermodynamics before turning his attention to the application of dynamical systems theory to problems in cell physiology, most notably complex bursting oscillations in pancreatic β-cells and Ca^2+^ waves in fertilized eggs [81,82].
Benno Hess (1922–2002). As director of a Max-Planck-Institute in Dortmund, Germany, Benno Hess directed a large group of biochemists and mathematical modellers exploring the molecular mechanisms of glycolytic oscillations in yeast cells [83].
Arthur T. Winfree (1942–2002). An engineer by training, Art Winfree turned his creative mind to the dynamics of oscillations and wave propagation in living organisms. His predictions of ‘phase singularities' in circadian rhythms and cardiac physiology revolutionized our understanding of these fields [84,85].
Rene Thom (1923–2002). The purest of pure mathematicians, Rene Thom was among the first persons to recognize the relevance of bifurcations of vector fields to temporal and spatial organization in living organisms [86,87].
Ilya Prigogine (1917–2003). The 1977 Nobel Prize in Chemistry was awarded to Ilya Prigogine for his fundamental insights on far-from-equilibrium thermodynamics, most notably on ‘dissipative structures' (temporal oscillations and spatial patterns) in living organisms [88].
Lee A. Segel (1932–2005). A world-renowned applied mathematician, Lee Segel made major contributions to the theory oscillations, pattern formation and wave propagation in living cells [89,90].
Reinhart Heinrich (1946–2006). A physicist who moved into biochemistry, Reinhart Heinrich combined modelling of specific cellular systems with a search for general principles. As one of the founding intellects behind metabolic control theory, he upset the paradigm of ‘rate-limiting' steps in biochemistry by showing, in quantitative detail, how flux control is distributed across all enzymes in a metabolic network [91].
Brian Goodwin (1931–2009). A Canadian émigré who got his PhD at the University of Edinburgh under Conrad Waddington, Brian Goodwin had a lifelong interest in development and evolution and was a leading figure in the renaissance of mathematical biology in the 1960s. His pioneering work on biochemical oscillators is reverberating to this day [92].
Christopher Zeeman (1925–2016). A leading English mathematician and early convert to Thom's theory of singularities of vector fields, Christopher Zeeman applied ‘catastrophe theory’ in creative ways to a wide variety of phenomena in biology, as well as other fields [93,94].
Rene Thomas (1928–2017). The Belgian biochemist Rene Thomas was an early proponent of ‘logical modelling' of biochemical reaction networks (i.e. modelling by Boolean functions). Early on he recognized the importance of negative feedback in generating biochemical oscillations and positive feedback in creating biological switches [95].
Gregoire Nicolis (1939–2018). A chemical physicist at the Free University of Brussels, Gregoire Nicolis led a team of talented and creative younger scientists in the application of Prigogine's abstract ideas about non-equilibrium thermodynamics and ‘dissipative structures' to real-world problems in chemistry, physics and biology [96,97].
George F. Oster (1940–2018). After an unusual start in the merchant marines and nuclear physics, George Oster studied biophysics and applied his massive intellect to a theoretical understanding of some of the toughest problems in molecular cell biology, including morphogenesis, pattern formation and molecular motors [98,99].
Garrett M. Odell (1943–2018). Trained in applied mathematics and theoretical mechanics, Garry Odell took an abrupt turn to mathematical biology, where he made many remarkable contributions to our understanding of chemotaxis, embryogenesis, molecular motors, gene regulatory networks and cytoskeletal mechanics [100].

## Data Availability

This article has no additional data.
